# pH Tunable Thin Film Gradients of Magnetic Polymer Colloids for MRI Diagnostics

**DOI:** 10.3390/polym12092116

**Published:** 2020-09-17

**Authors:** Sumera Khizar, Nasir M. Ahmad

**Affiliations:** Polymer Research Lab, School of Chemical and Materials Engineering (SCME), National University of Sciences and Technology (NUST), H-12 Sector, Islamabad 44000, Pakistan; sumera.phd@scme.nust.edu.pk

**Keywords:** magnetic polymer colloids, thin film gradients, layer by layer, MRI, lab-on-chip

## Abstract

Magnetic polymer colloids comprising of magnetite (Fe_3_O_4_) nanoparticles and Eudragit E100 were employed to fabricate thin film gradients and were investigated for in-vitro magnetic resonance imaging. Magnetic polymer colloids (MPC) and polyacrylic acid (PAA) with stimuli-responsive cationic and anionic functional groups respectively facilitate the formation of thin film gradients via layer by layer technique. The characteristics of films were controlled by changing the pH and level of the adsorbing solutions that lead to the development of gradient films having 5.5, 10.5 and 15.5 bilayers. Optical microscopy, scanning electron microscopy and magnetic force microscopy was carried out to determine the surface coverage of films. Surface wettability demonstrated the hydrophilicity of adsorbed colloids. The developed thin-film gradients were explored for in vitro magnetic resonance imaging that offers a point of care lab-on-chip as a dip-stick approach for ultrasensitive in-vitro molecular diagnosis of biological fluids.

## 1. Introduction

Smart nanomaterials based on biopolymers have gained significant importance in day to day life for their particular properties which make them suitable for a wide range of applications [1]. In addition to their biodegradability, biocompatibility, renewability and inexpensiveness, their adjustable physical and chemical properties in response to deliberately imparted external stimuli like temperature, humidity, pH, the intensity of light and electrical or magnetic fields has opened remarkable possibilities of manipulating them for diagnostic, therapeutic or even theranostic functions [2]. With the increasing developments in the fabrication approaches of nanotechnology devices, novel materials are being synthesized and studied for biomedical applications [3].

Currently, magnetically responsive systems possessing magnetic and biopolymers are the topic of great research due to their applications in theranostics, coatings, microfluidics and microelectronics fields [4]. Highly efficient magnetic responsive materials made up of biopolymers functionalized with magnetic nanoparticles are promising candidates in different fields of biomedicine such as a therapeutic agent for controlled drug delivery and hyperthermia and as contrast agents for magnetic resonance imaging (MRI) [5]. Iron oxide (superparamagnetic iron oxide Fe_3_O_4_ (magnetite)or γ-Fe_2_O_3)_ (maghemite))-based responsive systems show significant potential toward advancing personalized nanomedicine due to their unusual physicochemical and biological properties [6]. Magnetic contents, surface functionality and solubility of magnetic nanoparticles determine their interactions with biological systems. The surface coating is another important factor that directly affects colloidal stability, in vitro cell uptake, cytotoxicity, in vivo biodistribution, clearance and toxicity of well-characterized magnetic nanoparticles [7]. Efficiency and sensitivity of magnetic polymer colloids depend on the nature of the surface coating to facilitate disease diagnosis and treatment evaluation [8]. Much attention has been dedicated to the development of approaches to synthesize superparamagnetic, monodisperse, biocompatible for theranostic applications [9]. The properties and performance can be adjusted by surface modification using different coating and labeling agents immobilized on their surfaces, which has broadened their role in diagnosis applications. An ideal system incorporating biopolymers with magnetic nanoparticles offers effective switchability, lower aggregation and stability in the magnetization properties. Among various materials, magnetic polymer colloids are the most extensively used functional materials due to their unique physical, chemical, magnetic and biocompatibility properties nanoparticles [10].

Presently paramagnetic lanthanide complexes (largely gadolinium-based (Gd-based)) are mostly used as contrast agents in clinical MRI [11]. However, the usage of gadolinium-based contrast agents is limited due to the biological distribution and the retention of the residual gadolinium [12]. Consequently, magnetic polymer colloids containing in particular superparamagnetic magnetite nanoparticles have been widely studied as novel magnetic resonance imaging (MRI) contrast agents due to due to their excellent superparamagnetic properties, biocompatibility and biodegradability [13]. Recent advances have further improved the characteristics of contrast agents broadening their potential clinical applications in MRI. The magnetic colloids can be functionalized with specific functional groups to perform non-invasive imaging at the cellular and molecular level.

Considering the importance of iron oxide-based contrast agents, in the present work magnetic polymer colloids (MPC) have been used to fabricate thin film gradients by self-assembly multi-layer by layer technique (SAMU-LBL) at various pH combinations for their potential applications in the field of biomedicine. LBL technique is the most facile prevalent approach for preparing nanometer level molecular architectures using electrostatic adsorption between oppositely charged species onto solid substrate [14,15]. LbL assembly is a cyclical procedure in which a charged material is adsorbed onto a substrate and after washing, an oppositely charged material is adsorbed on top of the first layer. This constitutes a single bilayer with a thickness generally on the order of nanometers and the deposition process can then be repeated until the desired number multilayers have been assembled. The flexibility of the LBL technique not only lies in the range of suitable layer components but also in the wide range of substrates that can be used to deposit multilayers [16]. Layer-by-layer (LBL) assembly of oppositely charged polyelectrolytes on colloidal particles has recently been used to generate novel nano- and microcapsules with variable characteristics. Thin films of nickel ferrites and chitosan were fabricated by the LBL technique and investigated for their ability to generate an intensity difference using blood as a test liquid, however, T1 and T2 images were not explored in detail [17]. Using the same manual fabrication protocol, thin films were designed, fabricated and characterized but the magnetic behavior of the films was not tested [18]. The pH-responsive properties of the polyelectrolytes and polymers used in colloids offer an additional opportunity to tune the properties of films for many potential applications [19]. The properties of multilayer thin films comprising of magnetic colloids and oppositely charged weak polyelectrolytes strongly depends on solution pH [20,21].

**Figure 1:**
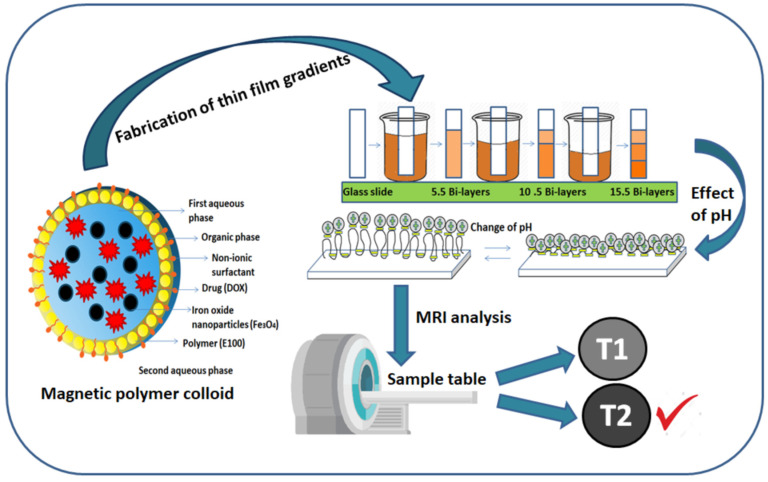
Schematic fabrication of magnetic polymer colloids thin film gradients and magnetic resonance imaging (MRI) diagnostics.

The present study employed the prepared magnetic polymer colloids to fabricate thin film gradients at various pH assembly conditions. These fabricated thin film gradients were characterized using different techniques to investigate the effect of pH on the surface wettability, surface roughness, thickness and film morphology. Also, this work explores these thin film gradients for in vitro magnetic resonance imaging. This technique offers a disposable lab-on-chip as a dip-stick approach that open new avenues to employ these novel bio thin film for various biomedical applications such as virus captures, analysis of biomacromolecules, DNA, RNA and so forth and magnetic colloids for various diagnostics and imaging applications including ultrasensitive in-vitro molecular diagnosis of biological fluids. The research work described here is novel and elaborate the fabrication of gradients films at various pH combinations. To the best of our knowledge, this research is the first work dedicated to the fabrication of gradient films using pH-responsive prepared colloids (MPC) and polyacrylic acid (PAA) for in vitro diagnostic investigations. Schematic of the fabrication of magnetic polymer colloids gradient thin films and MRI diagnostics is presented in Figure 1.

## 2. Materials and Methods

### 2.1. Synthesis and Characterization of Magnetic Polymer Colloids (MPC)

The synthesis and characterization of magnetic polymer colloids were similar as described in previous work [22]. Water in oil in water (W/O/W) emulsion was synthesized using magnetite nanoparticles (Fe_3_O_4_) prepared by coprecipitation. Magnetite nanoparticles of size 12 nm stabilized with nitric acid (HNO_3_, Sigma Aldrich) were dispersed in water along with the drug doxorubicin (DOX.HCL, Zheing jiang Pharmaceuticals, Taizhou, Zhejiang, China) as a first aqueous phase. Water in oil (W/O) was obtained by adding the first aqueous phase into an organic phase containing polymer Eudragit E100 (Evonik Industries, Darmstadt, Germany) dissolved in ethanol (Sigma Aldrich, Germany). The prepared W/O emulsion was then stabilized in the second aqueous phase containing surfactant Tween 80 (BDH supplies laboratory, UK) to obtain W/O/W emulsion. 

The prepared magnetic polymer colloids were characterized using different techniques. The size and morphology of magnetic colloids were observed by a scanning electron microscope (SEM, JEOL JSM 6490LA, Japan). Fourier transform infrared (FT-IR) analysis was carried out to study and identify chemical functional groups present in colloids with an FTIR spectrometer (Shimadzu 8400, Canada) in the range of 400–4000 cm^−1^. The surface charge, size distribution and polydispersity index (PDI) of obtained magnetic colloids was determined by zeta potential measurements by Malvern Zetasizer (Nano ZS, Malvern Instruments, UK).

### 2.2. Synthesis of Polyacrylic Acid

Polyacrylic acid was synthesized under an inert N_2_ atmosphere using free-radical polymerization of 10 g (0.14 M) acrylic acid monomer (Sigma-Aldrich, Germany) and 0.0270 g (0.1 mM) of potassium persulphate (K_2_S_2_O_8_, Sigma-Aldrich, Germany) water-soluble initiator in 90 mL water for 6 h at 60 °C. The synthesized polymer was further purified to remove residual liquids through freeze-drying and under vacuum drying until constant weight observed. The molecular weight of the prepared polymers was determined using viscosity measurements and found to be 24,500 g/mol [23]. The dried polymer was dissolved in water to make a 75% solution for further usage to fabricate thin film gradients.

### 2.3. Fabrication of Thin Film Gradients 

Thin film gradients were fabricated in two steps by layer-by-layer technique using a diluted solution of prepared magnetic polymer colloids (MPC) and 10^−3^ M solution of polyacrylic acid (PAA). The MPC solution was used as a positively charged solution and PAA solution as a negatively charged solution. Thin films were fabricated by alternate dipping of negatively charged glass slides (Fischer) in the solution of MPC and PAA having opposite charges. 

**Figure 2:**
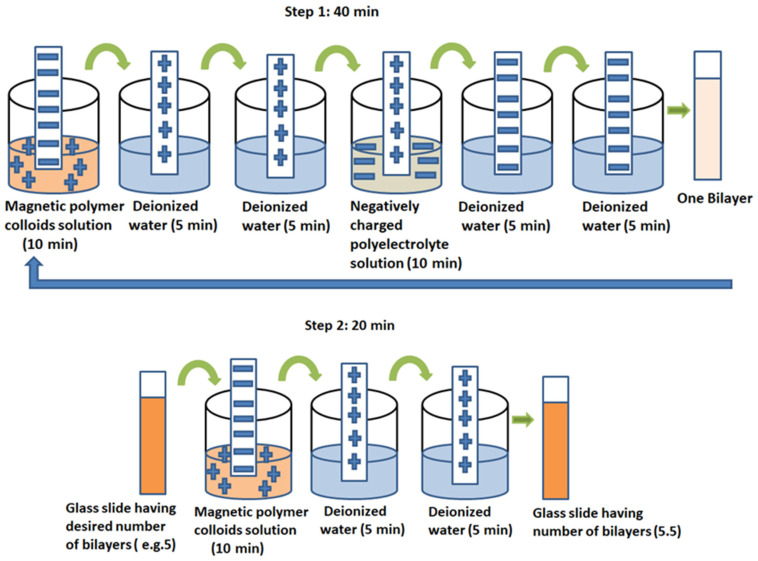
Schematic representation of fabrication of thin films using layer-by-layer technique.

In this technique, glass slides were first cleaned by overnight dipping in chromic solution (10 wt. % aqueous potassium dichromate: conc. H_2_SO_4_ 50:50 *v/v*) by which these acquire negative charges on their surface. In the first step, glass slides were first dipped in a positively charged solution of magnetic polymer colloids (MPC) for ten minutes and this resulted in the deposition of the first positive layer. This is followed by rinsing in ultrapure water twice for five minutes in separate water baths which removes all the unbound molecules from the surface. Then the glass slides were exposed to the negatively charged solution of polyelectrolyte (PAA) for ten minutes followed by rinsing in ultrapure water for five minutes in two water baths separately. This results in a single bilayer formation on the substrate that includes one layer of magnetic polymer colloids and one layer of polyelectrolyte. To study the characteristics of magnetic polymer colloids in thin films, after getting the desired number of bilayers (5, 10 and 15), in second step glass slides were dipped in a solution of magnetic polymer colloids again for ten minutes followed by rinsing in ultrapure water twice for five minutes in two separate water baths. This results in a single layer deposition of only magnetic polymer colloids (not a complete bilayer). Thus bilayers (5.5, 10.5 and 15.5) were deposited on a glass slide with a top layer of magnetic polymer colloids that acts as an active layer. The number of bilayers deposited on the substrate is determined by the number of cycles that the substrate undertakes in the first step. The whole procedure of the layer-by-layer technique is presented in Figure 2. The first step of single bilayer deposition is completed in 40 min and the second step in 20 min. To fabricate multilayers of bilayers, the whole process of dipping glass slide in alternate solutions of opposite charges is repeated without drying. Following this scheme, the desired number of bilayers can be deposited on the glass slide and dried in air. 

**Figure 3:**
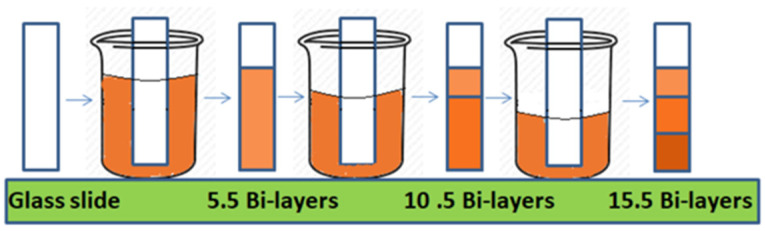
Schematic representation of fabrication of thin film gradients using layer by layer technique.

The gradients were made by changing the level of solutions, thus changing the exposed surface to the solution as shown in Figure 3. The level of the adsorbing solution was changed after the deposition of every 5.5 bilayers to make gradient films with 5.5, 10.5 and 10.5 bilayers on a single glass slide.

Films with three distinct regions with 5.5, 10.5 and 15.5 bilayers as gradients were obtained with the outermost layer of magnetic polymer colloids. Four types of gradients films were fabricated at four different combinations of pH (6/4), (5/5), (4/6), (3/7) (pH of MPC/pH of PAA) by varying the pH of magnetic polymer colloid (from 6 to 3) and polyelectrolyte solutions (from 4 to 7). The pH of the MPC/PAA was altered from 6/4 to 3/7 by using 1 M HCl and 1 M NaOH solution. Thin films gradients fabricated at different pH combinations had been given the following codes presented in Table 1.

### 2.4. Characterization of Thin Film Gradients

Thin film gradients were characterized to study the growth and morphologies using different techniques. Optical microscopy (Optika B-600 MET, Germany) was used to observe the surface coverage of thin film gradients. Scanning electron microscopy (SEM, JEOL JSM 6490LA, Japan) was used to investigate the surface morphology and distribution of magnetic colloids in different sections of thin film gradients. Optical profilometry (NANOVEA PS-50, USA) was used to measure the surface thickness and roughness of the prepared gradient. Magnetic force microscopy (MFM) was used to obtain contrast images of films to characterize the magnetic colloids at the nanoscale using atomic force microscopy (AFM, Joel JSPM-5200, Germany) in tapping mode with MFM hover mode under ambient conditions to observe the deposition of bilayers in each section. The surface wettability of the prepared film gradients was tested by Drop Shape Analyser DSA 25 (KRUSS Advanced 1.8.0.4, Germany) by measuring the contact angle of each section of gradient films by using the ADVANCE software installed in the drop shape analyzer.

### 2.5. In Vitro MRI Analysis

To analyze the in-vitro diagnostic capability of thin films of the prepared magnetic emulsion, MRI scans were carried out in the water by using a clinical MRI machine (GE SIGNA EXPLORER, USA) of 1.5 Tesla having FOV of 22 cm and aperture of 60 cm. MRI of thin films was also performed using T1 and T2 sequences with different values of MRI field echo (FE) sequences (Repetition time-TR and Echo time-TE). Glass micro slide was immersed in ultrapure water in a custom made polyacrylic cell. Then the cell is placed inside a magnetic coil to obtain MRI images. T1 and T2 weighted images with different values of MRI field echo (FE) sequences (TR and TE) to study signal intensity were acquired. Contrast images were analyzed and Region of interest (ROI) was calculated as a statistical tool K-PACS Workstation (Version 1.5). Water was used as a control to compare the contrast of colloids. The same region of interest (ROI) was taken in every section of films different number bilayers and uncoated regions were taken as a reference. The intensity data was measured in Hounsfield units based on the number of pixels contained within the ROI. The relative intensity of each section was compared were measured by taking the mean values of the intensity.

## 3. Results and Discussion 

### 3.1. Characterization of Prepared Magnetic Polymer Colloids

**Figure 4:**
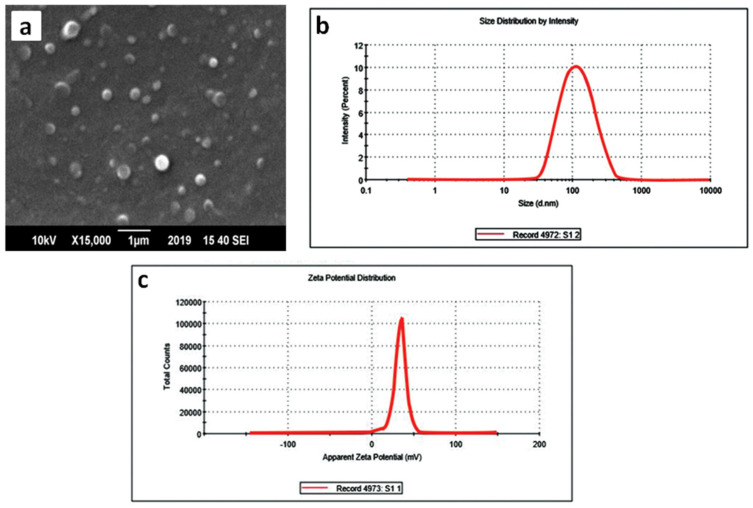
(**a**) Scanning electron microscopy (SEM) image; (**b**) Particle size distribution and (**c**) Zeta potential curve of prepared magnetic polymer colloids.

The magnetic polymer colloids employed to fabricate films contain a magnetic core of magnetite nanoparticles having positively charged functional groups on their surface [22]. The average size and zeta potential of the colloids were found to be 108 nm and +32.8 mV, respectively which indicate significant stability of the colloids displayed in Figure 4. The positive charge on the colloids was due to the protonation of the tertiary amine group present in polymer Eudragit E100. The polydispersity index (PDI) of 0.235 indicates that the particles are in uniform size distribution.

### 3.2. Effect of pH on Adsorbing Solutions

Change in pH of adsorbing solutions on variation in structure and properties of layer-by-layer assembled multilayer thin films was demonstrated by employing positively charged magnetic polymer colloids and negatively charged PAA through variation in the pH of solutions. The purpose to change the pH of colloids and polyelectrolyte was to investigate its influence on the deposition of films on a glass substrate. The characteristics of films that include surface wettability, surface roughness, film morphology and bilayer thickness experience significant variation with changes in pH of a polyanion PAA and polycation MPC [20]. 

**Figure 5:**
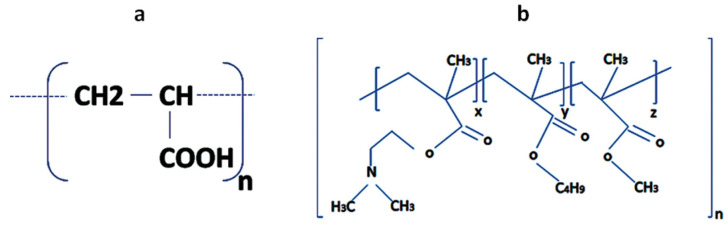
(**a**) Chemical structure of polyacrylic acid (PAA); (**b**) Chemical structure of Eudragit E100.

Polyacrylic acid (PAA) is a pH-responsive, anionic, weak polyelectrolyte and easily soluble in water and its physical properties are highly reliant on pH due to the presence of an ionizable carboxylic functional group (-COOH) as shown in Figure 5a. It is used as a negatively charged polyelectrolyte for the fabrication of gradient films. By changing the pH of polyacid, one can tune the density of linear charges of the polymer chain. Polyacrylic acid responds to pH with changes in chain conformation, passing from coiled to extended flat conformation [24]. At pH less than 4, precipitation occurs due to protonation of the carboxylate group, which reduces polymer solubility in water [25]. By increasing the pH of PAA by the addition of a base, ionization occurs and polymer expands into an open coil conformation resulting in clear and concentrated solution [26]. The linear charge density along weak polyelectrolyte backbones is a function of pH. The charge density on the polymer also changes with pH, which affects the growth of films and results in a wide diversity of film characteristics [27]. An increase in pH causes coiled and loopy PAA to stretch out [28], dissociation of the functional group occurs and more of the polymer chains will become charged. At low pH, polymer presents a low charge density, where the number of attachments to the surface is much lower as compared to a highly charged polymer at a relatively higher pH [29]. Thus at low pH, PAA has loopy conformation having less exposed charges leading to a decrease in adsorption of cationic colloidal particles in multilayers. There is a loose and loopy polymer adsorption takes place at the pH of the PAA deposition solution near to the pKa value of 4.5 [16].

Magnetic polymer colloids having Eudragit E100, which is a cationic pH-responsive coating polymer with dimethylaminoethyl ammonium functional groups having low solubility at pH neutral and a high solubility at pH below 5 [30]. Eudragit E100 is prepared by the polymerization of acrylic and methacrylic acid or their esters, both of these polymers are biodegradable, non-toxic and synthetic displayed in Figure 5b. Eudragit E100 is soluble in organic solvents and insoluble in water in its unprotonated form near to its pKa value of 7–7.3 [31,32]. At pH 5, Eudragit E100 is partially protonated that contributes to its swelling behavior [32]. By decreasing the pH of MPC, it becomes more positive due to the protonation of dimethylaminoethyl ammonium groups suggesting an increase in the polymer surface charge density. Under extreme acidic conditions, the polymer degrades due to the hydrolysis of polymer side chains containing alkyl esterified groups, affecting their capability of coating [33]. Above pH 5, the polymer becomes permeable and swellable leads to slow adsorption of polymer to the interface [34]. The pH of colloidal suspension also affects the deposition characteristics of multilayers. 

Negatively charged carboxylate groups in PAA showed strong coordination with positively charged tertiary amine groups present on colloid surfaces that result in film formation on the substrate. It is interesting to note that the influence of the pH of MPC on film growth is less than that of PAA in the pH range investigated.

### 3.3. Optical Microscopy of Thin Film Gradients

**Figure 6:**
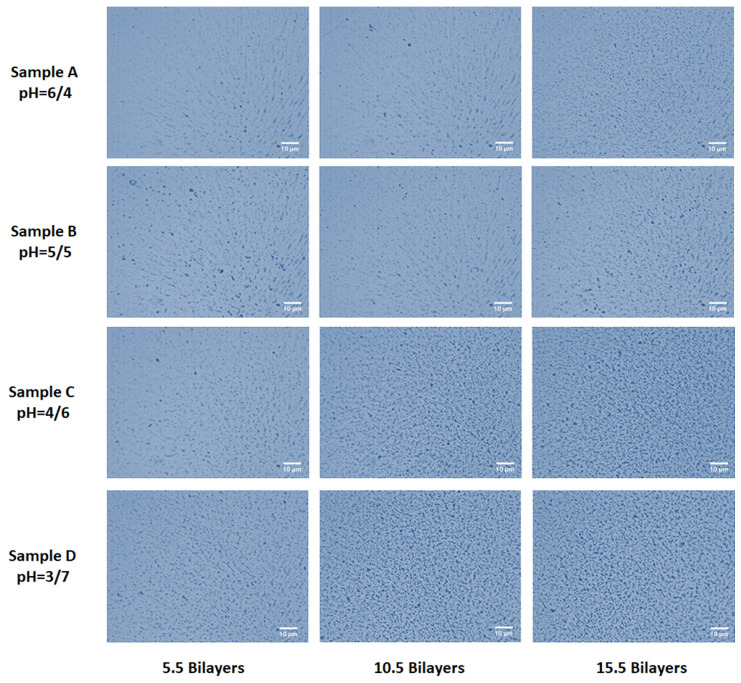
Optical microscopy of thin film gradients of samples A, B, C and D fabricated at various pH combinations.

Optical microscopy was performed to observe the consistency in different sections of gradient thin films of magnetic polymer collides. Figure 6 shows the results that darker areas increase along the surface with the increase in the number of bilayers in the case of all samples. As the number of bilayers increases from 5.5 to 15.5, there was more deposition with some agglomeration of colloids [35].

An increase in the surface coverage was observed in the images of optical microscopy of thin film gradients that as the pH of MPC/PAA was varied from 6/4 to 3/7. There is a more uniform distribution of colloids in films in sample D at pH combination of 3/7, which decreases gradually with the increase of pH of MPC from 3 to 6. The opaqueness of the films increases due to the random deposition of colloids on the substrate in a non-uniform manner with the increasing pH of PAA from 4 to 7. Films are appeared cloudy in sample A that showed the least deposition of MPC/PAA films. In samples B and C, distributions of colloids were in between the two extreme pH combinations studied of 6/4 and 3/7 [36].

### 3.4. Optical Profilometry of Thin Film Gradients

The conformation of the adsorbing layers and the previously adsorbed layer determine the bilayer thickness of the films [37]. Table 2 summarizes the thickness of the prepared films at various pH of PAA and MPC. The thickness of MPC/PAA bilayers decays exponentially with increasing pH of the PAA solution. Bilayer thickness was varied by changing the pH of adsorbing solutions from few to tens of micrometers. There was significant growth of films, as can be seen from the thickness profile as the pH of PAA increases and the pH of MPC decreases. The coating thickness decreases due to the adsorption of thin and flat underlying PAA adsorbing layer at high pH. These changes in thickness can be related to change in the conformation of polymer with increasing pH [24]. The thickness of adsorbed bilayers varies from 0.7 µm to 16.5 µm as the pH of dipping solutions was adjusted. The lower thickness of gradient films can be attributed to the desorption of the polymer [38]. It is observed that polymer chains were adsorbed as flat chain conformations at high pH with high ionized form and more loopy structures at low pH because of relatively low ionization. As the pH of PAA increases, more colloidal nanoparticles were adsorbed on the adsorbed PAA layer due to high charge density resulting in thick bilayers. MPC has less effect on the thickness of bilayers as compared to PAA. The amount of polyelectrolyte adsorbed from low to medium charged state changes the conformation of the adsorbed layer [39]. The film surface roughness and thickness increase with the increase in the number of deposited bilayers due to more adsorption of polyelectrolyte and magnetic colloids from 5.5 bilayers to 1.55 bilayers. 

**Figure 7:**
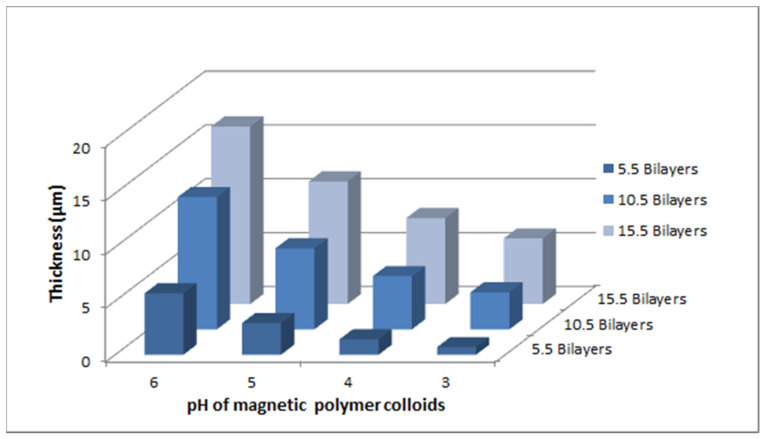
Thickness profile of thin film gradients (A, B, C and D) versus pH of magnetic polymer colloids.

Figure 7 shows the average incremental thickness of bilayers fabricated by sequential adsorption of cations (MPC) and anions (PAA). The results revealed that the deposition of bilayers was significantly influenced by the pH of individual dipping solutions. By varying the pH, it is possible to tailor the thickness of deposited films. Thin layers were observed that were due to flat chain conformation of the polymer of high pH value and thicker attributed to its loopy structures [28].

**Figure 8:**
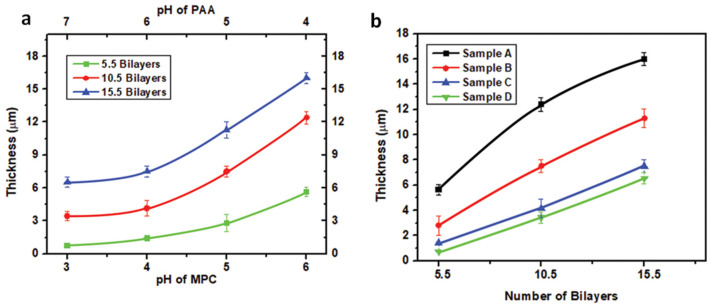
Thickness of the prepared thin film gradients. (**a**) Effect of the combinations of pH of MPC/PAA and (**b**) Effect of the number of bilayers.

Figure 8a,b are the representation displayed the thickness versus combinations of pH of MPC/PAA and thickness versus the number of bilayers. Layer by layer deposition proceeds in a linear and reproducible manner up to 15.5 bilayers. It is observed, at all pH combinations that with the increase in the number of bilayers the thickness increases exponentially. 

### 3.5. Surface Coverage in Thin Film Gradients

**Figure 9:**
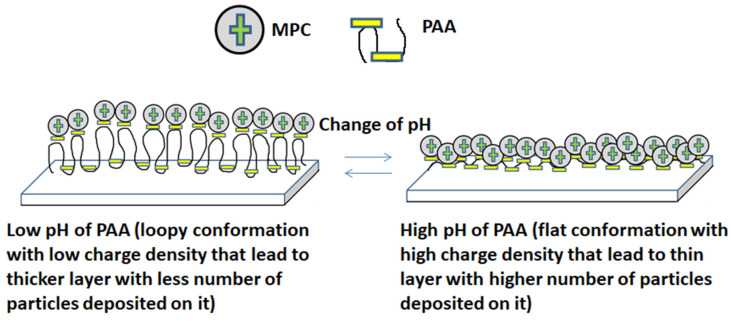
A schematic illustration of the behavior of PAA at low and high pH and sequential adsorption of magnetic polymer colloids (MPC).

The self-assembly includes the adsorption of MPC onto an oppositely charged substrate from a dilute aqueous solution that leads to charge reversal of the surface. As the pH of MPC decreases, there is a complete charge reversal that results in a significant growth of thin films. The charge on the underlying layer would determine and explain the number of charges adsorbed on the surface. Since positively charged MPC is attempting to adsorb onto a negatively charged PAA layer for sequential adsorption of bilayers. At a high value of pH, high charge density favoured thin bilayer growth and adsorption of densely packed nanoparticles as shown in Figure 9. At low charge density, the number of attachment points is lower and less exposed as compared to the highly charged polymer that leads to irregular adsorption of colloids [40]. Complete charge reversal of bilayers is essential after each deposition step for the successful growth of films that was observed at pH combination of 3/7. There was dense adsorption of colloidal particles in the assembly of bilayer as can be seen from SEM images at pH 3/7 which showed deposition of a large number of particles, closely spaced, due to high charge density of adsorbed layers. In the case of low charge density, there was a negligible growth of films that was observed at a pH combination of 6/4 because of the low ionization of PAA. PAA is adsorbed in the highly charged form at pH above 5. Thus, the amount of MPC adsorbed to a surface increases with increasing charge density since more charge is required to compensate and balance the high charge density of the surface. This novel approach provides control over the amount of charges adsorbed onto the surface by changing the pH of solutions.

**Figure 10:**
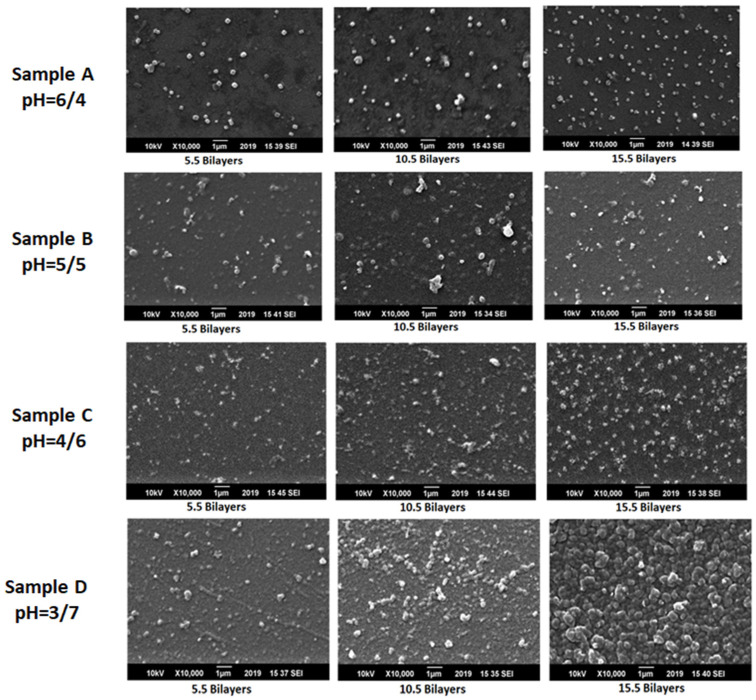
SEM micrographs of thin film gradients of samples A, B, C and D fabricated at various pH combinations.

It is also observed that the surface coverage increases with a number of bilayers from 5.5 to 15.5 which confirmed the optical microscopy results. At 5.5 bilayers, the surface has less number of adsorbed particles as compared to 10.5 and 15.5 bilayers. There is a non-uniform distribution of colloids in all the samples which can be overcome by increasing the number of washing steps to cover more surface. The substrate–polycation and dipping time of substrate in the dipping solution may also affect the growth of films. Non-uniform deposition during layer by layer deposition of colloids was maybe due to the irregular adsorption of anionic PAA on cationic colloidal particles and also on the surface of the substrate. The anomalous adsorption of oppositely charged cationic and anionic particles enhances the agglomerations because of the electrostatic interaction between them. Electrostatic interactions between oppositely charged colloids and polyelectrolyte played an important role in the growth of films. Therefore, in thin film gradients, several regions with agglomerated particles were observed. Sample D shows a high degree of agglomeration of particles that increases with the increase in the number of bilayers which can be due to the high charge density of polyelectrolyte. The agglomeration increase with the increase and decrease of pH of polyelectrolyte and magnetic colloids respectively. Sample A has the most uniform distribution of colloids as compared to the films fabricated at other pH combinations due to the presence of few attachment points on the polyelectrolyte chain. The morphology of the adsorbed particles was not very clear but can be assumed nearly spherical from the images. Lower surface coverage was observed in sample A as compared to other samples. In sample B and C there was relatively more surface coverage with colloidal particles had taken place with agglomeration as compared to sample A. Figure 10 illustrated the SEM images of samples with 5.5, 10.5 and 15.5 bilayers. An interesting observation regarding the growth of thin-film gradients that one can tune the characteristics of films by controlling the pH of the dipping solutions without any significant change in shape and size. Thus magnetic polymer colloids retain their properties after fabrication and can be useful for in vitro biomedical applications such as MRI.

### 3.6. Surface Wettability of Thin Film Gradients

**Figure 11:**
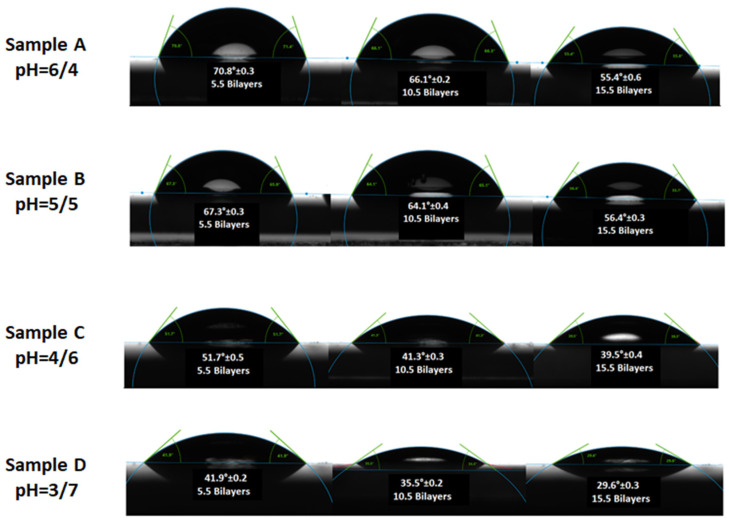
Contact angle measurement of thin film gradients at various pH combinations.

In the sequential adsorption process, the ability to control the wettability of assembly of bilayers via pH adjustments offer an advantage in creating functional thin films for specific applications. Variations in contact angle were observed by controlling solution pH in a layer by layer adsorption process of colloids and polyelectrolyte, which determines the hydrophilicity of the films [41]. Figure 11 represents the measurement of contact angles for the fabricated thin film gradients and surface energy values were summarized in Table 3. The change in wettability of thin films gradient depends on the number of bilayers, the chemical composition of the adsorbed layer, hydrophilicity of functional groups and level of the interpenetration of the outermost layer into the previously adsorbed layer [42]. Hydrophilicity of thin films increases with the increase of pH of PAA from 4 to 7 due to the interpenetration of the underlying layer polymer layer into the outermost layer [43]. The hydrophilicity of the gradient films was due to more deposition of particles at pH 3/7 and extended conformation of polymer showing its ability to interact more with water. Sample D has the most wettable surface at pH combination of 3/7 with a contact angle of about 29.6°. Sample A is a relatively less hydrophilic surface with a contact angle of about 70° for 5.5 bilayers which may be due to the hydrophobic nature of Eudragit E100 and fewer number particles adsorbed on the substrate. Sample B and D have intermediate hydrophilicity between two extreme pH combinations investigated in the present work.

At each pH combination, gradient films were fabricated having 5.5, 10.5 and 15.5 bilayers. The contact angle of thin film gradients decreases along the substrate with an increase in the number of bilayers from 5.5 to 15.5. This decrease was due to the increase in surface roughness of films that determine the wettability of surface [44]. A regular periodic decrease in contact angle values with the increasing number of bilayers was observed in the gradient thin films in all samples that may be attributed to the adsorption of a large number of particles as observed in SEM images. As the number of bilayer increases from 5.5 to 15.5, more charges get adsorbed on the surface, which leads to a decrease in contact angle. 

The contact angle analysis in Table 3 revealed that all the films are hydrophilic with a contact angle less than 90° which shows that these are capable of good interaction with water molecules which is important for biomedical applications like MRI. Low contact angle indicates that liquid will spread over the surface and surface is favorable for wetting whereas high contact angle means the liquid will produce a droplet by reducing contact area and the surface is not favorable for wetting [45].

The surface energy of the solid (substrate) was also calculated by using the results of contact angle measurements. The Young equation that relates contact angle to the surface energy is expressed as Equation (1) [46].

**Figure 12:**
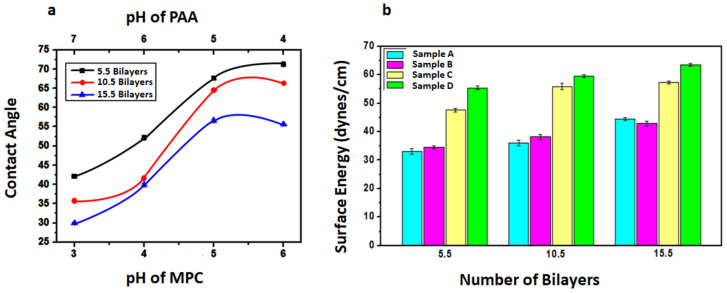
(**a**) Contact angle of bilayers of fabricated thin film gradients versus various pH combinations of MPC/PAA and (**b**) Surface energy versus the number of bilayers of samples (A, B, C and D) fabricated at various pH.

(1) γS=γSL+ γLcosθ,

where γS and γL are the surface energies of solid and liquid respectively, γSL is the solid-liquid interfacial energy and θ is the contact angle. γL and θ can easily be determined to calculate γS and γL by using different theoretical models [45]. The surface energy value helps to predict the wettability of the solid by a certain liquid. The surface energy of the solid γS can be estimated using the Fowkes equation [γS = 0.25γL(1 + cosθ)^2^] [47] in which one considers the interaction of liquid by only its polar components. As the contact angle increase, surface energy decreases that corresponds to the low wettability of the surface. Thus the surface energy is enhanced with a low contact angle. With the increase in the number of bilayers in all the samples, the hydrophilicity of the films increases, this indicates its ability to hold water molecules on the surface [48]. The values of contact angle and their corresponding surface energies are given in Table 3. As can be seen clearly that as the contact angle decreases with the increase of the number of bilayers in all samples due to an increase in surface roughness and their corresponding surface energy increases [49]. The surface energy is an important parameter that will determine not only the change in hydrophilic properties of films but also other properties like adhesion of water molecules to the surface and frictional forces at the interface [50]. Figure 12a represents the variation of contact angle measurement with the change of pH and Figure 12b increase of surface energy with an increase in the number of bilayers for the fabricated thin film gradients.

### 3.7. Quantitative Analysis of the Topography of Thin Film Gradients by MFM

**Figure 13:**
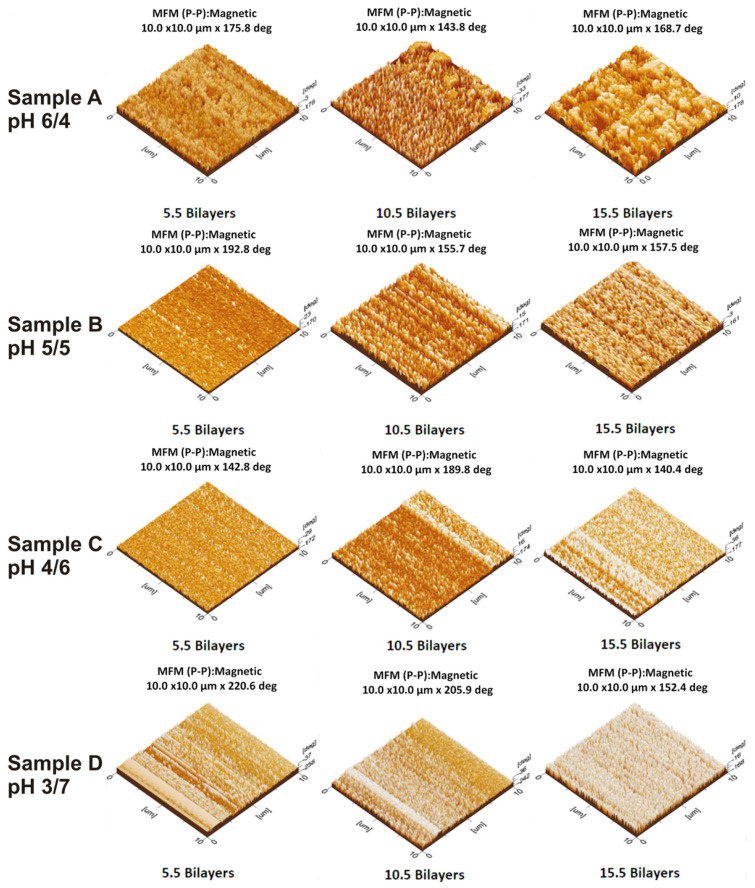
Magnetic force microscopy (MFM) images of thin film gradients of samples A, B, C and D fabricated at various pH combinations.

Surface morphology and roughness of the prepared films over the glass substrate were studied by magnetic force microscopy (MFM). Figure 13 shows MFM images of prepared thin film gradients. By MFM, one can able to detect and localize magnetic nanoparticles in films at the nanoscale. AFM probe was brought near a sample that interacts with magnetic domains on the surface. MFM detects local magnetic interactions by measuring deflections of the tip as it scans over the sample. The MFM images show the characteristics pattern with dark bright MFM contrast indicating magnetic domains. The individual magnetic nanoparticles cannot be detected by MFM because the magnetic field is directly proportional to the diameter of the particles and thus very small. MFM contrast was generated by the interaction between the MFM probe and the magnetic field close to the sample surface. These images showed the distribution of magnetic particles as well as the gradients. The negative values of phase shifts indicate attractive interaction of the tip with magnetic nanoparticles than with the substrate and can be used to characterize the nature of the interaction of the tip and sample and nature of the sample itself [51]. Contrast generated along the substrate is enhanced with the increase of a number of bilayers due to the increase in electrostatic tip-sample interactions due to the presence of magnetic nanoparticles in the samples. As the number of bilayers increases, brighter features were higher and the increase in density can be seen through the contrast caused by the magnetic properties of the surface being characterized. Agglomeration of particles in the form of clusters was successfully imaged in each section of bilayers, in particular films with 15.5 bilayers. MFM phase shift increase with the increase of the number of bilayers in all samples. Dark bright contrast in MFM images was due to the aggregation of magnetic nanoparticles and the existence of magnetic domains [52]. As the number of particles deposited increases as in sample D, bright features become more prominent. The samples with few particles like samples A and B have less interaction with the tip, thus agglomerates behave as single dipoles [53]. Sample C has a contrast between samples B and D. The nonlinear deposition of magnetic colloids when a number of bilayers are small was due to the dominant interaction between substrate and polycation. An increase in the amount of polyelectrolyte adsorbed increases the adsorption sites for colloidal particles and ultimately increases the contrast in MFM images.

### 3.8. MRI Studies of Thin Film Gradients

In the present work, MRI characteristics of thin film gradients prepared from magnetic polymer colloids and PAA at various pH combinations were investigated. All the samples of thin film gradients were exposed to a strong magnetic field in an MRI machine. A radiofrequency pulse was applied causing atoms at spin and relaxes. This relaxation emits energy which is detected by an MRI scanner and is converted into image mathematically. Two types of scans were carried out to obtain T1 and T2 weighted images. T1 was the spin-lattice relaxation in which longitudinal components of the magnetization vector, exponentially recovered to its initial value. On the other hand, T2 was characterized by spin-spin relaxation time in which the transverse component of the magnetization vector exponentially decayed towards its equilibrium value of zero in MRI. T1 and T2 weighted images with different values of MRI field echo (FE) sequences (TR and TE) were acquired to study signal intensity. T2 weighted images were acquired at a constant TR value of 5000 ms but variable TE conditions (TE-45, 75, 105, 135 and 165 ms) and T1 at TE value of 20 ms but variable TR conditions (TR-420, 520, 620, 720 and 820 ms). All micro slides of the samples were tested at room temperature by placing them in polyacrylic cells (three at a time) using deionized water as a test liquid. The cell was then placed inside the MRI machine. The MRI images, obtained in axial mode (along the *z*-axis), with a slice thickness of 4.5 mm and a gap of 1 mm, were analyzed using the host software, SV 25 (Version 2.8.0.101) available on the MRI machine and statistical tool K-PACS Workstation (Version 1.5). MRI images were recorded and equal region of interest (ROI) was drawn manually to obtain average intensity data based on a number of pixels within ROI.

**Figure 14:**
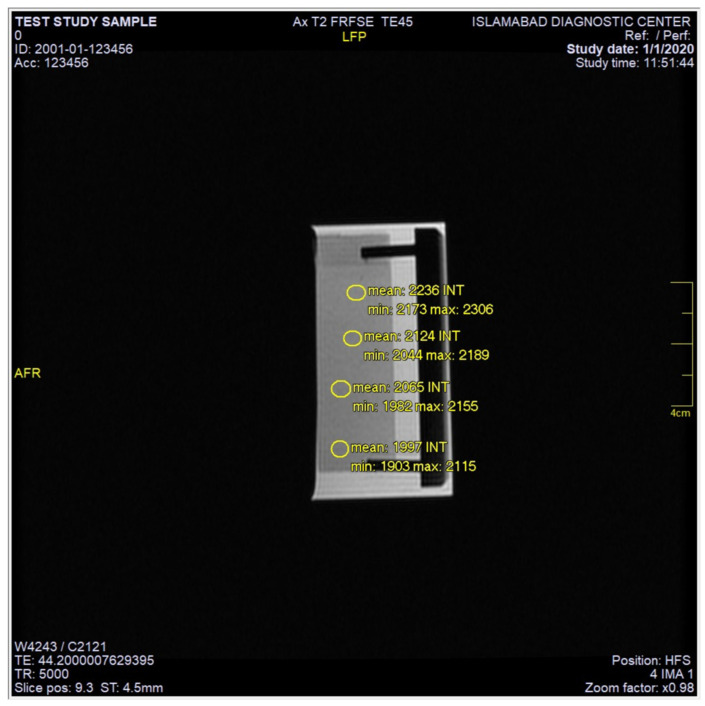
A representative MRI image of a thin film gradient. The figure illustrates the mean signal intensity values of region of interest (ROI) chosen in different sections of the film. The ROI intensities show a decreasing trend with an increase in the number of bilayers along the gradient.

A representative T2 weighted image of thin film gradients is given in Figure 14, where a marked difference in signal intensities of four distinct regions (uncoated, 5.5, 10.5 and 15.5 bilayers) was observed. A region of interest (ROI) of equal areas were manually drawn, starting from the uncoated region and then of 5.5, 10.5 and 15.5 bilayers. Each ROI value is the mean of three signal intensities values along with the standard deviation.

**Figure 15:**
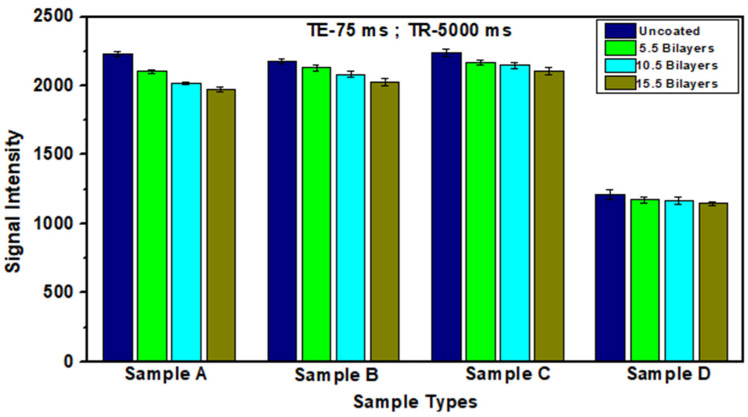
Mean signal intensity graph of thin film gradients of samples A, B, C and D fabricated at various pH combinations for TE 75 ms and TR = 5000 ms.

Figure 15 shows the comparison of signal intensity data for the fabricated films at different pH combinations for a single value of TE (75 ms) and TR (5000 ms). Differences in intensity as well as in the number of bilayers within a sample were visible in T2 weighted images. The results of ROI in different sections of the thin film gradients of all samples showed a decrease in signal intensity with the increase in the number of bilayers. A decrease in signal intensity along the gradient in every sample (from uncoated onto 15.5 bilayers area) revealed that the films can generate negative contrast. The intensity values decrease with the increase in particle concentration which is evident from optical microscopy and SEM results. The prepared films have good contrast ability which increases along the gradient as compared to the uncoated part. The uncoated region was taken as a reference to compare the difference between the contrasts generated by the gradients. Variation of pH of PAA and magnetic polymer colloids have affected the hydrophilicity and particle deposition of magnetic colloids on the substrate, thus ultimately relaxivity of the films [54]. T1 and T2 weighted images are related to the interaction of magnetic polymer colloids with hydrogen nuclei of water molecules in the sample [55].

As the number of bilayers increases in samples, signal intensity decreases may be attributed to the increase in surface wettability of films [56]. A difference in signal intensity was observed among uncoated, 5.5, 10.5 and 15.5 bilayer parts in the gradient films prepared at different pH combinations. The T2 intensity variation with the number of bilayers also reveals the change in relaxation rates due to energy exchange between protons in water molecules. The increase in the concentration of deposited magnetic colloids along the gradient leads to a decrease in signal intensity from higher to lower values, in each sample. The hydrophilic nature of PAA also allows proximity of water molecules to the substrate on which particles were deposited leading to a shortening of T2 relaxation time [54].

The mean signal intensity of four samples of every bilayer section was calculated along with the standard deviations and plotted against for a particular TE and TR value. There is a consistent decreasing trend observed at all TE sequences for every sample. As the number of bilayers increases, signal intensity decreases that confirmed the generation of iron oxide concentration along the length of the substrate. As the number of deposited particles increases with the number of bilayers, it corresponds to the increase in the iron oxide content present in the bilayer section. An increase in iron content ultimately decreases the signal intensity along the gradient in all the samples investigated.

Sample A at pH 6/4 has the highest intensity difference among different sections of bilayers. Intensity difference among uncoated, 5.5, 10.5 and 15.5 bilayers decreases with an increase and decrease of pH of PAA and MPC respectively. Sample B and C have ROI values between samples A and D. The signal intensity data of all samples revealed that at the same value of TR and TE the most lowering of signal intensity was observed in sample D prepared at pH combination of 3/7 due to high hydrophilicity of gradient films as compared to gradients films fabricated at other pH combinations. The lowest intensity at pH 3/7 was due to the deposition of a greater number of particles as shown in SEM images. However, a less difference in mean signal intensity among various sections of bilayers was observed in sample D due to the dense growth of bilayers. Agglomeration of magnetic particles due to self-assembly also affect the capability of the films to generate the contrast upon interaction with water molecules that form the basis of detection [57].

T1 weighted images were also obtained at different TR values keeping TE (20 ms) constant. In contrast to T2 results, there are relatively less changes for T1 weighted intensity images. The size of magnetic colloids also confirmed them as T2 contrast agents because exceedingly small magnetic nanoparticles show effective T1 contrast [58,59]. T1 also requires proximity of water molecules to the contrast agents but the results of present formulation and PAA used in the fabrication of thin-film gradients do not seem to affect T1 relaxivity.

The MRI results indicate the potential of the developed gradient films of magnetic polymer colloids as negative contrast agents for future point-of-care lab-on-chip in vitro molecular surface-based imaging via MRI technique. These films can further be investigated by using bioanalytes to observe the visualization of cells. This would be highly useful in the future to develop a technique for efficient imaging of bioanalytes such as red blood cells, white blood cells and platelet. Fabrication of gradient films can also be carried out by using magnetic colloids conjugated or functionalized with specific biomarkers such as nucleic acids (DNA and RNA), proteins, autoantibodies and cells, which further enhance the cellular visualization [60]. MRI data of thin films magnetically engineered thin film gradients can also be reported for cancerous and healthy cells. Such surface-based thin-film gradients eliminate the requirement to inject magnetic materials to an object and involvement of living organisms under examination.

## 4. Conclusions

The current study presents the novel potential of stimuli-responsive thin film gradients of magnetic polymer colloids. Fabrication of thin film gradients was carried out by adsorption of oppositely charged polyelectrolyte and prepared colloids on a substrate. The diagnostic capability of film gradients was investigated and it is noted that a significant T2 contrast was generated along the gradient. Thus the developed films and can be successfully employed as a simple reproducible approach as a lab-on-chip device. This work is an important step towards future research for point of care diagnostics. The pH responsiveness of films provides control over the surface coverage of the substrate and ultimately regulates the interaction of biological analytes for diagnostic purposes. In the future, these pH-responsive thin film gradients can be used to manipulate various interfacial phenomena such as drug delivery. These multifunctional thin film gradients can be used in a wide range of biomedical applications.

## Figures and Tables

**Table 1:** Codes of different samples prepared at various pH combinations.

Sample Codes	A	B	C	D
**pH of MPC/pH of PAA**	6/4	5/5	4/6	3/7

**Table 2:** Thickness measurement of samples A, B, C and D at various pH combinations.

Sample Codes	pH of Emulsion	pH of PAA	Thickness (µm)		
			5.5 Bilayers	10.5 Bilayers	15.5 Bilayers
**A**	6	4	5.63 ± 0.4	12.4 ± 0.5	16.5 ± 0.5
**B**	5	5	2.81 ± 0.7	7.51 ± 0.5	11.3 ± 0.7
**C**	4	6	1.42 ± 0.07	4.15 ± 0.7	7.49 ± 0.7
**D**	3	7	0.734 ± 0.05	3.44 ± 0.4	6.53 ± 0.4

**Table 3:** Contact angles and surface energy of 5.5, 10.5, 15.5 bilayers of samples A, B, C and D fabricated at various pH combinations.

Number of Bilayers	Sample A		Sample B		Sample C		Sample D	
	Contact Angle θ(°)	Surface Energy γS (dynes/cm)	Contact Angle θ (°)	Surface Energy γS (dynes/cm)	Contact Angle θ(°)	Surface Energy γS (dynes/cm)	Contact Angle θ (°)	Surface Energy γS (dynes/cm)
**5.5**	70.8° ± 0.6	33 ± 0.944	67.3° ± 0.3	34.6 ± 0.5	51.7° ± 0.5	47.5 ± 0.5	41.9° ± 0.2	55.4 ± 0.5
**10.5**	66.1° ± 0.2	35.9 ± 1	64.1° ± 0.4	38.1 ± 0.7	41.3° ± 0.3	55.9 ± 1	35.5° ± 0.2	59.6 ± 0.5
**15.5**	55.4° ± 0.3	44.5 ± 0.5	56.4° ± 0.3	42.9 ± 0.9	39.5° ± 0.4	57.3 ± 0.5	29.6° ± 0.3	63.5 ± 0.5
